# Gleanings from Our Exchanges

**Published:** 1872-02-01

**Authors:** 


### Gleanings from Our Exchanges.

### MEDICAL DEPARTMENT CHICAGO RELIEF AND AID SOCIETY.

The following in relation to the medical department of the Chicago Relief work, we extract from the First Special Report of the Chicago Relief and Aid Society:

The Committee on Sick, Sanitary and Hospital Measures is composed of men representing fairly the medical profession of the city. Dr. H. A. Johnson is the Chairman. Provisions for medical relief have been made as follows:

VISITATION.

The city has been divided into districts and sub-districts, with the same boundaries and the same offices as those of the Superintendents of Distribution.

To each of these divisions a Medical Superintendent and a sufficient number of visiting physicians have been appointed. Their duties are defined as follows:

*First.* Each visiting physician will establish an office in connection with the depot of distribution in his district.

*Second.* He will, at a specified hour, morning and evening, visit the office and answer such calls as may be left by the Superintendent of Distribution, the visitors, and the Medical Superintendent of the district.

*Third.* He will supply himself with a case and medicines for the use of those only who are the proper subjects of relief by this Society.

*Fourth.* He will affix to each prescription that he may send to the dispensing chemist, his signature, with a statement that this prescription is on account of the Chicago Relief and Aid Society.

*Fifth.* He will especially examine into the sanitary condition of his district, the quantity and quality of food, clothing, dwellings, etc., and all matters having a bearing upon public or private health.

*Sixth.* He will report daily to the Medical Superintendent of his district, the name, age, sex and nativity of each patient, with the name of the disease, result of treatment, number of visits to each patient, and such other information as the Medical Superintendent may, from time to time, require.

*Seventh.* Each Medical Superintendent will have the immediate direction of the medical service and sanitary interest of his district, and will be held responsible for the faithful performance of this work. He will assign visiting physicians to sub-district, and require of them a daily report of their work. These reports he will condense and present weekly to the Committee. He will admit patients to hospital, and in cases of emergency, visit patients at their homes.

The General Superintendent has directed visitors to report all cases coming to their knowledge requiring medical attendance, and the person in charge of each office has such reports at all times in readiness for the medical officer of the district when he calls. All possible aid is given the medical officer of the district, and he is allowed free access to the office and books of the Society at all times.

MEDICAL DISPENSARIES.

In addition to this provision for the visitation of the sick at their homes, dispensaries have been established at convenient points, where such patients as are able to apply in person for advice are treated, and where medicines are dispensed upon the prescription of any physician certifying that his services in the case are gratuitous. In the North Division of the city there is now only one of these institutions; another will be opened as soon as the need of it shall be evident. In the West Division there are three, and in the South Division two. Medicines are also dispensed and out-patients treated at all of the hospitals.

HOSPITALS.

For the relief of such patients as cannot safely be treated in their homes or quarters, and who cannot apply at a dispensary, hospital accommodations have been provided. Fortunately the principal hospitals of the city were in the unburned district. Arrangements have been made with all these institutions by which patients are received on account of this Society, without charge for medical and surgical attendance, nursing and general care, the Society furnishing only medicines, rations and furniture for such relief patients as may be received on its account. These hospitals are as follows:

The Providence Hospital, located just beyond the northern limits of the city. The Women's and Children's Hospital, formerly located on North State street, but now temporarily at No. 598 West Adams street. This is mainly a lying-in hospital. The Chicago Eye and Ear Infirmary, under the care of Dr. E. L. Holmes, before the fire on Pierson street, in the North Division, now at 579 West Adams street. St. Luke's Hospital, on Indiana avenue, between Fourteenth and Sixteenth streets. The Scammon Hospital, on Cottage Grove avenue, near Twenty-ninth street. The Mercy Hospital, corner of Calumet avenue and Twenty-sixth street, and the County Hospital, Arnold street, near Eighteenth street.

In addition to these accommodations, the Committee are building a hospital in the burnt district of the North Division. The plan is essentially that of the United States army hospitals. Hospitals are also being constructed in connection with the barracks in the West and North Divisions of the city. Patients are admitted to hospitals upon the order of the medical officers of the Chicago Relief and Aid Society, the Sanitary Superintendent of the Board of Health, and the County Physician.

Supplies to hospitals and dispensaries are issued upon requisitions endorsed by the chief medical officer of the institution, and approved by the Chairman of the Committee on Sick, Sanitary and Hospital Measures.

The dispensaries and hospitals report daily to the chairman of this Committee the number of patients treated, number of deaths, number of recoveries, and, as often as required, the names of relief patients under treatment. With the daily reports from the visiting physicians, and these reports from hospitals and dispensaries, the committee will be able to give at any time the name and address of every patient treated, and at the close of their work the result of the case.

BURIALS.

Arrangements have been made with the county authorities by which, at a small cost to the relief fund, all who may die while under the care of this department will be furnished a coffin and hearse to any of the cemeteries in the vicinity of the city. Orders for such burial are given by Dr. Jno. H. Rauch, of the Board of Health, Dr. B. C. Miller, County Physician, or Dr. H. A. Johnson.

HOW TO OBTAIN MEDICAL RELIEF.

To obtain medical relief it is only necessary to make application to some one of the Superintendents of Distribution, to a visitor of that bureau, or to a Medical Superintendent. The offices and office hours of the physicians of the Society will be found in the directory at the end of this report.

SANITARY REGULATIONS AND CONDITION.

The sanitary questions connected with houses and barracks have been carefully considered, and the suggestions of this Committee have been adopted by the Committee on Shelter. The barracks are subject to a careful, daily inspection by sanitary officers, and regulations best calculated to maintain health are rigidly enforced. The statistics thus far indicate that these quarters are probably more healthy than those occupied by the same class of tenants before the fire. Up to the present time, November 30, only one death has occurred among a population of five thousand in barracks.

For the last four years our city has experienced a singular immunity from small-pox. We can hope to maintain this only by the same measures hitherto used, namely, vaccination and revaccination. This has been made compulsory in the barracks, and all of our citizens have been urgently advised to submit themselves to the same operation. The returns from hospitals, dispensaries and visiting physicians, show that, to the date of this report, about five thousand patients have been cared for by the medical officers of this Society.

A circular has been prepared and issued, earnestly inviting the co-operation of our citizens in providing for the sick proper nourishment, delicacies, and such care as cannot be given by the physician. It is believed that material aid will thus be secured to the Committee in the administration of medical relief.

This department is indebted for valuable assistance to the Board of Health, and especially to the Sanitary Superintendent, who has given his personal attention to the sanitary arrangements and police of the houses and barracks.

The medical force is made up of men prominent in the profession, and earnest and conscientious in the discharge of their duties; and with the above provisions, it is believed that help will be brought within the reach of all, and none will suffer for anything that humanity, guided by educated art, can do for them.

### MARRIAGE *v.* CELIBACY.

M. Bertillon has read a paper at the Academy of Medicine, at the sitting of November 14, entitled "Influence du mariage sur la duree de la vie et sur les maladies intellectuelles ou morales," which is itself only a portion of an article on marriage intended for the "Dictionarie encyclopedique des sciences medicales." He maintains the healthy influence of conjugal association as compared with that of celibacy. He proves his assertions by statistical facts drawn from the statistics of France, Holland, and Belgium. They embrace a period of ten years.

According to his figures, if we consider the masculine sex, it will be seen that from twenty-five to thirty years of age, 1,000 married men furnish 6 deaths; 1,000 bachelors, 10 deaths; and 1,000 widowers, 22 deaths. From thirty to thirty-five years of age, 1,000 married men furnish 7 deaths; 1,000 bachelors, 11½ deaths; 1,000 widowers, 17½ deaths. From thirty-five to forty years of age, 1,000 married men furnish 7½ deaths; 1,000 bachelors, 13 deaths; and 1,000 widowers, 17½ deaths. And so on, in a series of tables for all ages, the married man has always the advantage of the single man. But the widower has a still greater mortality than the bachelor. How can this be explained? In the case of young men married under the age of twenty, the statistics show it to be the reverse of salutary. Out of 8,000 young men married before twenty, their mortality was, before marriage, only 7 per 1,000; after marriage, it was 50. This is a constant result that is found in Paris, Belgium and Holland. Everywhere young married people, from eighteen to twenty years of age, die as fast as old people of from sixty to seventy years of age. From these facts we must conclude, with Hufeland, "that the premature use of the genital organs is the most sure means of inoculating old age." This astonishing mortality can be attributed to no special malady, but to a general enervation which, without doubt, renders them an easy prey to any disease.

With respect to the feminine sex, we find the same advantage of marriage over celibacy, though not quite so marked. Up to the age of thirty no perceptible advantage is derived from marriage. From thirty to thirty-five the mortality is 11 per 1,000 for single women, and only 9 per 1,000 for married women. This difference increases up to fifty-five. Thus, from fifty to fifty-five years of age, 1,000 wives furnish only 15 or 16 deaths; whilst as many single women or widows furnish 26 or 27. This advantage remains very notable beyond that age, diminishing but little. But, in France under twenty-five years of age, and in Paris under twenty, marriage is far from favorable, but even injurious, as in the case of the masculine sex. The mortality of young girls from fifteen to twenty is 7.53 per 1,000. The mortality of wives of the same age is 11.86. The mortality of girls from twenty to twenty-five is 8.32; of wives, of the same age, 9.92. Without doubt, the cause of so great an augmentation of mortality in married women, from twenty to twenty-five, is pregnancy, which, disappearing towards the age of forty years, permits the privilege of married women to recover its equilibrium. The deadly effect of widowerhood on men has been seen; it is not less deadly in the case of women, but ceases to be so after thirty; and after forty-five more single women than widows die.

The influence of marriage on criminality is not less striking. Whilst the criminality of single men for crimes against persons is 100, for married men it is only 49, and 45 for offences against property. The criminality of widows and widowers approaches that of celibacy. Relative to suicide, the author concludes, from his researches, that this cause of death is reduced one-half by marriage; and he adds, that it has the same for madness.—*The Doctor*, *Jan.* 1, 1872.

Vaccination Papers.—Small-pox having entered our hitherto healthy town, there has been a great demand for vaccination; and I have, at times, found difficulty in keeping up a regular supply of lymph. To meet this, I have devised a new method of lymph preservative; it is most portable, and very certain in its effects. Taking a sheet of common sized (*i. e.*, cream-laid) note paper, I paint it with fresh lymph taken from the vesicle with a fine camel-hair pencil. The lymph soon dries; and the paper is ready for use. When required, a minute piece may be cut out or torn off the sheet, and, after having been slightly breathed upon, should be stuck upon the freshened surface. If the paper be required to be kept for any length of time, it should after being charged, be covered with a thin coating of white of egg. Isinglass will not do, as it cracks when dried—*Preston*—*Brit. Med. Jour.*, *Dec.* 23, '71.

Complete Excision of the Astragalus and Os Calcis.—Dr. T. G. Morton, of Philadelphia (*Am. Journ. Med. Sciences*), presented a case of excision of these bones, at a meeting of the College of Physicians and Surgeons. A very perfect recovery followed, both as regards motion in the new joint and in the usefulness of the foot, which was shortened about one inch.

